# Mitigating effect of gallic acid on zinc oxide nanoparticles and arsenic trioxide-induced spermatogenesis suppression, testicular injury, hormonal imbalance, and immunohistochemical changes in rats

**DOI:** 10.1007/s00210-024-03228-y

**Published:** 2024-06-27

**Authors:** Amany Behairy, Mohamed M. M. Hashem, Khaled Abo-EL-Sooud, Abeer E. El-Metwally, Ahmed M. Soliman, Samar M. Mouneir, Bayan A. Hassan, Yasmina M. Abd-Elhakim

**Affiliations:** 1 Department of Physiology, Faculty of Veterinary Medicine, Zagazig University, Zagazig, 44519 Egypt; 2 Department of Pharmacology, Faculty of Veterinary Medicine, Cairo University, Giza, 12211 Egypt; 3 Pathology Department, Animal Reproduction Research Institute, Giza, 3514805 Egypt; 4 Pharmacology Department, Faculty of Pharmacy, Future University, Cairo, 11835 Egypt; 5 Department of Forensic Medicine and Toxicology, Faculty of Veterinary Medicine, Zagazig University, Zagazig, 44519 Egypt

**Keywords:** Zinc oxide nanoparticles, Arsenic trioxide, Gallic acid, Proliferating cell nuclear antigen, Inducible nitric oxide synthase, Testis

## Abstract

The current study compared the effects of incorporated exposure to arsenic trioxide (As) and zinc oxide nanoparticles (ZnONPs) on male reproductive hormones, oxidative stress, and inflammatory biomarkers in adult rats to each metal alone. A defensive trial with gallic acid (GA) has also been studied. A total of 60 adult male Sprague Dawley rats were categorized into six groups: control, GA (20 mg/kg), ZnONPs (100 mg/kg), As (8 mg/kg), ZnONPs with As, and GA concurrently with ZnONPs and As at the same previous doses. The regimens were applied for 60 days in sequence. Current findings showed significant weight loss in all study groups, with testicular weights significantly decreased in the As and combined groups. Testosterone, follicular stimulating hormone, and luteinizing hormone serum levels were also considerably reduced, while serum levels of estradiol increased. Inducible nitric oxide synthase (iNOS) immunoexpression was significantly upregulated while proliferating cell nuclear antigen (PCNA) was downregulated. Moreover, there was a significant elevation of testicular malondialdehyde, reduction of testicular superoxide dismutase, and glutathione peroxidase with disruptive testes, prostate glands, and seminal vesicle alterations in all experimental groups with marked changes in the combined group. Additionally, the present results revealed the protective effects of GA on ZnONPs and As adverse alterations in rats. GA enhanced sperm picture, oxidant status, and hormonal profile. Also, it modulates iNOS and PCNA immunoexpression and recovers the histoarchitecture of the testes, prostate glands, and seminal vesicles. Ultimately, GA may be a promising safeguarding agent against ZnONPs and As-induced disturbances to reproductive parameters*.*

## Introduction

Zinc (Zn), the second-most important trace element, is plentiful in the human body (Vickram et al. 2021). Zinc oxide nanoparticles (ZnONPs) are extensively employed nanomaterial in sunscreen products, personal hair and skin care products, ceramic products, paints, coloring agents, food storage, ceramics, and drugs (Alosaimi et al. 2024; Khan et al. 2024). Due to their modified surface area and particle size distribution, ZnONPs exhibit higher cytotoxicity when compared to conventional Zn (Chen et al. 2023). In this regard, a large number of previous studies in murine models demonstrated that ZnONPs exposure reduced sperm motility and altered its morphology (Hong et al. 2022; Ziamajidi et al. 2023). Additionally, in the recent in vivo and in vitro trials of Chen et al. (2023), ZnONP accumulation disturbed the formation of the blood-testis barrier (BTB) and synthesis of growth factors, which in turn suppressed spermatogonia proliferation and promoted their apoptosis. The cytotoxicity of ZnO NPs in the testis is mediated by oxidative stress, cell death, and other mechanisms (Tang et al. 2019; Pinho et al. 2020).

Arsenic (As), a naturally occurring metalloid, is a widely recognized environmental contaminant (Hu et al. 2019). Arsenic is abundant in surface waters, soil, and groundwater (Rahaman et al. 2021). Inorganic As levels in urban air range from 0.02 to 0.03 μg/m^3^, while 0.1–50 μg/m^3^ near industrial sources (Mukherjee and Valsala Gopalakrishnan 2023). It is highly used in manufacturing wood, glass, pigments, and colorants (Gliozzo and Burgio 2022). Exposure to As leads to accumulation in the testes and epididymis, causing structural damage, oxidative stress, inflammation, autophagy induction, and apoptosis (Kumar et al. 2022). Additionally, exposure to As damages spermatocytes, impairs sperm integrity, and reduces the quantity, viability, and motility of spermatozoa (Han et al. 2020a). As toxicity may also be associated with diminished gonadotropin secretion, testosterone production, and compromised steroidogenesis (Han et al. 2020b). Besides, it promotes excessive ROS production, disrupts the oxidative surroundings of testicular tissues, and boosts malondialdehyde (MDA) levels (Olfati and Tvrda 2021). Besides, As dramatically lowered epithelial height, tubular width, and luminal diameter in rodents, leading to testicular spermatogenic degeneration (Guvvala et al. 2017). Arsenic exposure also triggered the inducible nitric oxide synthase (iNOS) expression (Shao et al. 2018). Moreover, As exposure could damage DNA in sperm cells via free-radical mediated responses (Zhang et al. 2021).

Gallic acid (GA) is a polyphenol identified in numerous natural sources, like green tea, grapes, mango, oak bark, and multiple berries (Gholamine et al. 2021). Compared to many polyphenolic compounds, GA acid displays the highest antioxidant potential and wide bioavailability (Badhani et al. 2015). After oral administration, nearly 70% of GA is absorbed and then excreted via urine (Konishi et al. 2004). Increasing the plasma levels of GA also required repeated dosage (Ferruzzi et al. 2009). GA is garnering attention for its anti-radical and selective apoptosis-inducing properties (Hosseinzadeh et al. 2019). This latter effect is caused by the molecule’s prooxidant properties (Yang et al. 2020). Additionally, GA improves phase-2 enzymes, comprising quinone reductase and glutathione S-transferase (GST), as well as antioxidant enzymes like glutathione reductase (GR), GPx, and CAT (Topkara et al. 2022). Hence, GA has several crucial physiological effects, including anticancer, anti-inflammatory, antioxidant, anti-diabetic, anti-fungal, anti-viral, and anti-allergic activities (Abdel-Moneim et al. 2018; Mehraban et al. 2020; Singla et al. 2020). GA’s ability to block the NF-κB pathway confers its regulatory impact against inflammatory responses. It downregulates the expression of pro-inflammatory cytokines, including interleukin-1β (IL-1β), TNF-α, interleukin- 8 (IL-8), and interleukin-6 (IL-6) (Hosseinzadeh et al. 2019).

Consequently, the current study attempted to examine the impaired effects of ZnONPs and/or As on sperm characteristics, antioxidant status, hormonal imbalance, and testicular tissue changes, individually and in combination. Furthermore, to our knowledge, the protective effects of GA on male reproductive problems resulting from combined exposure to ZnONPs and As have not been reported. Hence, in this work, we investigated how GA could protect the testes of rats exposed to ZnONPs and As from inflammation and free radicals.

## Materials and methods

### Chemicals and reagents

Alpha Chemika in Cairo, Egypt, supplied ZnONPs, a nanopowder with serial no. Al 4235 A00010 has a molecular weight of 81.39 g/mol, a purity of 99.5% based on metal traces, and an average particle size of 30 ± nm. It was dissolved in Egyptian SEDICO Company distilled water. Also, arsenic trioxide (As_2_O_3_, serial no.0498 00100.2H_2_O, MW = 197.84, and 99% purity) and gallic acid (serial no.2256 00025, C_7_H_6_O_5_. H_2_O, MW = 188.14) were supplied by Alpha Chemika in (Cairo, Egypt). The Sigma Company manufactured all additional compounds employed during the investigation and were of the highest analytical grade.

### Animal model and experimental scheme

**Fig. 1:**
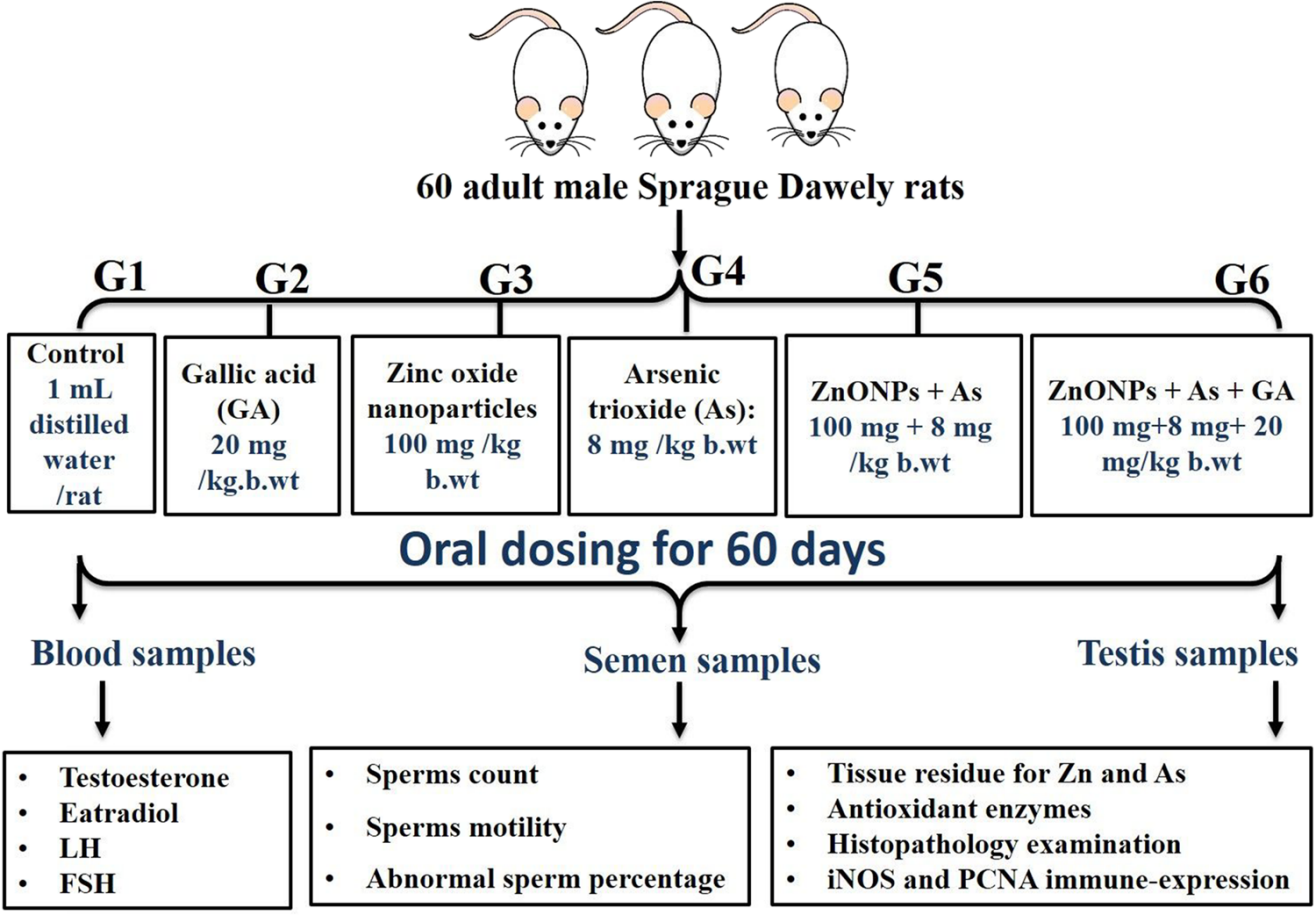
Experimental protocol, sampling, and estimated parameters

Sixty adult Sprague Dawley rats (male, average weight 160.33±1.03) were attained from the National Research Center breeding division (Giza, Egypt). All rats were kept in clean, adequately ventilated stainless-steel mesh cages with a 12-h light-dark cycle and relative humidity ranges from 50 to 60%. Wood-shaving bedding was employed to keep the cages dry. For the experiment, rats were provided endless access to tap water and typical rodent food. Two weeks before the testing, rats were subjected to the experimental settings. The rats were weighed and allocated randomly to one of six groups (*n* = 10 each) as follows (Fig. 1):

- G1 (the control group) received 1 mL of distilled water/rat per day
- G2 (the GA group) was orally administered 20 mg/kg b.wt. GA dissolved in distilled water (20 mg/6.25 mL) (Yigitturk et al. 2017).
- G3 (the ZnONPs-exposed group) orally administered 100 mg ZnONPs /kg b.wt (Hussein et al. 2016). ZnONPs were suspended in distilled water and sonicated for 5 min before dosing.
- G4 (the As-exposed group) orally administered 8 mg As /kg b.wt (Pei et al. 2019).
- G5 (the ZnONPs+As group) orally co-administered ZnONPs and As at the same previous doses.
- G6 (the ZnONPs+As+GA-treated) co-orally administered ZnONPs, As, and GA at the same previous doses.

For 60 days, each treatment was delivered orally once daily between 8 and 10 a.m. by a feeding needle (16 gauge). For the following reasons, we decided that a 60-day treatment period would have the greatest impact on spermatogenesis: The average length of a rat’s seminiferous cycle is 12.5 days, and mammals, including rats, go through approximately 4.5 cycles. After accounting for spermatocytogenesis, meiosis, and spermiogenesis, the overall time required for spermatogenesis is around 48–52 days (Perrard et al. 2016). In rats, the epididymal transit of spermatozoa takes around 1 week (Fernandez et al. 2008). Thus, a 60-day exposure would properly be the duration of dosing to assess spermatogenesis. All dosing volumes were adjusted weekly based on the rats’ body weight changes.

### Serum and testicular sample collection

One intramuscular injection of a combination of 5 mg/kg b.wt xylazine and 50 mg/kg b.wt ketamine hydrochloride was administered to the rats to anesthetize them on day 61 of the investigation. After that, the rats were weighed. To collect blood samples from the rats, a sterile glass capillary tube was used to penetrate the rat’s retro-orbital venous plexus. For hormonal tests, blood was taken into a standard centrifuge tube to collect serum. Immediately following the decapitation of each rat, the testes were extracted, freed of any connective tissues or fat, and then weighed. After measuring the testis’s mass, the relative testicular weight was calculated using the following formula: Relative testis weight is equal to testis weight divided by body weight and then multiplied by 100 (Kumar and Devi 2018). Three groups of testis samples were formed. The first group of specimens was preserved in 10% buffered formalin for histopathological and immunohistochemical assessment. A tissue homogenizer (Potter-Elvehjem, Thomas Scientific, Swedesboro, NJ, USA) was used to homogenize 0.5 g of testicular samples from the second group in cold potassium chloride. It was then centrifuged at 664 ×g for 10 min at 4°C to obtain the homogenate to analyze testicular enzymes and antioxidants. As and Zn concentrations were measured by keeping the last group at 4°C.

### Semen assessment

After the rats were decapitated, the cauda epididymis was quickly removed and sliced into small pieces using sterile scissors. Then, 2 mL of physiological saline (warmed at 37°C) was added. The suspension that emerged was evaluated for sperm concentration, motility, and abnormalities. The epididymal suspension (one drop) was placed on a clean hemocytometer and heated to 37°C before being inspected under a microscope (Olympus Soft Imaging Solutions GmbH, Munster, Germany), at 40 *×* magnification. Within 2–4 min, we examined 200 epididymal sperm throughout many microscopical fields. A subjective scoring system with a 0 to 100% range was employed to estimate the proportion of movable sperm cells (Slott et al. 1991). To count the sperm, a hemocytometer chamber slide was used. The spermatozoa were inactivated by treating the slide with four drops of 40% formalin after being diluted (1:4) in normal physiological saline. The formula used to estimate the concentration of sperm cells in a 1 mL sample was as follows: n (the number of sperm in 0.1 mm^3^ of diluted semen) × dilution factor × 5 × 10 × 1000 (Brito et al. 2016). To prevent sperm cell interconnection and boost sperm count for exact numbers, a 25-fold dilution factor was used. The Filler (1993) protocol was employed for estimating the percent of unusual sperm in eosin/nigrosine-stained smears. Ten µL of formalin-mixed sperm solution was cautiously merged with nigrosine and a 5% eosin solution (15 µL) on a glass slide. The smears were then ready air-dried, and explored under a 400 *×* magnification microscope. One hundred spermatozoa were chosen at random and investigated for any abnormality in three key areas (head, neck/mid-piece, and tail).

### Hormones evaluation in serum

With a sensitivity level of less than 0.06 ng/mL and a detection range of 0.13 to 25.6 ng/mL, the rat testosterone ELISA kits (catalog number CSB-E05100r) obtained from Cusabio Biotech Company (Houston, TX, USA) were used to measure serum testosterone level. A rat estradiol ELISA kit with a sensitivity of less than 4.38 pg/mL and a detection range of 12.35–1000 pg/mL was purchased from Kamiya Biomedical Company in Seattle, WA, USA. The KT-15332 rat FSH ELISA kit (with a detection range of 2.47–200 ng/mL and a sensitivity of 1.11 ng/mL) and the KT-21064 rat LH ELISA kit (with a detection range of 0.37–30 ng/mL and a sensitivity of 0.153 ng/mL) were also available from Kamiya Biomedical Company in Seattle, WA, USA.

### Oxidative stress biomarkers analysis

The activity of glutathione peroxidase (GPx) in the testicular homogenate was measured using enzyme-linked immunosorbent assay (ELISA) kits from Elabscience Biotechnology Co., Ltd. (Houston, Texas, USA). The kits had a sensitivity of 18.75 pg/mL and a detection range of 31.25–2000 pg/mL. To evaluate the superoxide dismutase (SOD) activity in the testicular homogenate, an ELISA Kit developed by Cusabio Biotech Co., Ltd. of Houston, Texas, USA (Cat No. CSB-E08555r) was utilized. The kit has a detection range of 7.8–500 U/ml with a sensitivity of less than 1.95 U/ml. Biodiagnostic kits (Cairo, Egypt) (Cat. No. MD 25 29) were used to determine the MDA level in the testicular homogenate.

### Examination of Zn and As residues in testicular tissues

The testicular specimens were chewed in microwaves with nitric acid and 30% hydrogen peroxide. An ICP-OES model 5100 (Agilent, Santa Clara, CA, USA) was used to analyze the sample for Zn and As concentrations. The amplitude of every reading in the series was compared to a blank and three Merck Company (Darmstadt, Germany) standards. The instrument values were verified, and the accuracy and reliability of the metal measures were confirmed by comparing them to a quality control sample containing the National Institute of Standards and Technology (NIST) standard reference material for metal traces.

### Histopathological and histomorphometric investigation

The testis, seminal vesicle, and prostate gland that had been treated with formalin were thoroughly dried, xylene-cleared, and paraffin-blocked. Following the protocol outlined by Suvarna et al. (2018), a single, representative, four-micron thickness cross-section of the collected organs/animal was stained with hematoxylin and eosin. Using a light microscope (Olympus Soft Imaging Solutions GmbH, Munster, Germany), an experienced non-biased pathologist blindly examined the sections at different magnifications. The pathologist blindly examined the slide twice and compared the results. For morphometric analysis of the testicular tissue sections, five-round or nearly round randomly selected and non-duplicated tubular sections (50 sections/10 animals/per group) were captured at 10× and 40×/animal (100 images/group). The images were used for assessing the testicular morphology including the height of the germinal epithelium, the diameter of the seminiferous tubules, the diameter of the tubular lumen, the thickness of the tunica propria, and the thickness of the testicular capsule (Babazadeh and Najafi 2017).

### Immunohistochemistry evaluation

#### iNOS immunohistochemistry

Tissue sections were deparaffinized, immersed in PBS, and treated for 10 min with 0.3% hydrogen peroxide in PBS to inactivate endogenous peroxidase. PBS was used to wash the sections before normal horse serum (10%) was applied. The anti-iNOS antibody utilized was a mouse monoclonal (Cat. 905-385 Neomarkers Inc., Fremont, CA, USA). At a 4°C overnight incubation, sections were probed with iNOS (1:100) antibodies. Normal IgG was used in place of the primary antibodies for negative control sections to demonstrate the specificity of the antibodies. Sections were rinsed in PBS before being incubated with biotinylated secondary antibodies (Cat.85-9043 Secondary kits Invitrogen Histostain plus kit, Broad spectrum, CA, USA) at room temperature for 30 min and after addition of the avidin-biotin-peroxidase complex. Immunostaining was done by a DAB kit (Spring Bioscience Inc., CA, USA). The sections were prepared for mounting after being dried and counterstained with Mayer Hematoxylin. The sections were examined by a light microscope (Olympus Soft Imaging Solutions GmbH, Munster, Germany). Brownish cells positive for iNOS by immunohistochemical staining were analyzed. To estimate iNOS expression as a percentage, we counted positive cells (at least 500 in each testis) in 5 randomly chosen fields (400×) containing 100 cells each and then divided the sum by 5 (Sohrabi et al. 2017).

#### PCNA immunohistochemistry

Anti-PCNA primary antibodies (Clone PC 10, DAKO A/S, Denmark) were used in this study. Dilutions of 1:50 were made of the main antibody in Tris-buffered saline. Following an overnight incubation at 4°C, the tissue sections were probed with the primary antibody. A commercial avidin-biotin-peroxidase detection system was used to monitor the binding of the primary antibody. We used a mouse monoclonal antibody instead of the primary antibody as a negative control. After that, diaminobenzene (DAB) was used as the chromogen, and hematoxylin was used as the counterstain on the slides. Light microscopy (×400) was used to check for PCNA immunostaining in the tissue sections. A random selection of microscopic fields was made. Each rat’s testicular changes were measured across five fields on each of the ten slides. Each seminiferous tubule’s PCNA was calculated as a ratio of the number of immunolabeled cells to the total number of basal cells (Tousson et al. 2011).

### Statistical analysis

The Shapiro-Wilk test assessed data normality, whereas the Levene test was applied to identify variance homogeneity. The data was then analyzed via one-way ANOVA and the post hoc Tukey test, which established significance when *P* < 0.05 was achieved. Data were presented as means ± SE. Prism 7.0 GraphPad (Graph-Pad, San Diego, CA, USA) was implemented for the statistical analyses.

## Results

### Effects on body weight, weight gain, and testicular weight

As shown in Table 1, the single administration of ZnONPs or As displayed a significant (*P* < 0.05) reduction in final body weight by 6.25% and 6.92%, respectively, and body weight gain by 16.60% and 17.73%, respectively, than the control group. Compared to the control group, the ZnONPs+As group indicated a marked reduction in final body weight (10.37%) and weight gain (30.57%). Body weight and weight gain change were significantly increased (*P* < 0.05) in the ZnONPs+As+GA-treated group than the As+GA–co-exposed group and were lower by 3.19% and 10.56%, respectively, than the control group.

**Table 1:** Effect of gallic acid (GA) on body weight change, gonadosomatic index, and sperm characteristics of adult male Sprague Dawley rats exposed to zinc oxide nanoparticles (ZnONPs) and/or arsenic trioxide (As) for 60 days

Estimated parameters	Experimental groups					
	Control	GA	ZnONPs	As	ZnONPs + As	ZnONPs+As+GA
Initial Body weight (g)	162.33±1.03	161.67±1.18	161.33±0.94	160.67±0.47	163.33±2.36	163.67±1.31
Final body weight (g)	250.67^b^±2.95	257.67^a^±0.62	235.00^d^±2.55	233.33 ^d^ ±2.36	224.67 ^e^ ±0.24	242.67 ^c^ ±2.87
Body weight change (g)	88.33 ^a^ ±3.86	96.00 ^a^ ±0.82	73.67 ^b^ ±2.95	72.67 ^b^ ±2.62	61.33 ^c^ ±2.25	79.00 ^b^ ±4.02
Testes weight (g)	2.30 ^ab^ ±0.04	2.43 ^ab^ ±0.16	2.60 ^a^ ±0.16	1.73 ^b^ ±0.05	1.67 ^c^ ±0.05	2.13 ^b^ ±0.05
Gonadosomatic index (%)	0.92 ^b^ ±0.01	0.95 ^b^ ±0.06	1.11 ^a^ ±0.07	0.74 ^c^ ±0.03	0.74 ^c^ ±0.02	0.88 ^b^ ±0.03
Sperm count (sp.cc/mL × 125 × 10^4^)	68.67^b^ ± 0.88	75.00^a^ ± 1.73	55.33^d^ ± 2.03	56.00^dc^ ± 2.08	33.67^e^ ± 2.73	62.00^c^ ± 2.08
Sperm motility (%)	89.33^a^ ± 0.67	93.33^a^ ± 1.67	46.67^cd^ ± 2.40	50.00^c^ ± 2.89	41.67^d^ ± 4.41	66.67^b^ ± 1.67
Sperm abnormalities (%)	17.00^d^ ± 1.15	12.67^e^ ± 0.66	32.62^b^ ± 0.83	32.01^b^ ± 0.83	39.76^a^ ± 1.72	27.50^c^ ± 1.26

Means within the same row carrying different superscripts are significantly different at* p* < 0.05. The values shown are means ± SE. *n* = 10

The relative and absolute testicular weights showed a significant (*P* < 0.05) increase in the ZnONPs group by 20.65% and 13.04%, respectively, but a marked reduction in the As group by 24.78% and 19.57%, respectively, than the control group. A marked (*P* < 0.05) affection of the ZnONPs+As group in the relative and absolute testicular weights was 19.57% and 27.39% compared to the control group. Yet, the ZnONPs+As+GA combined group did not significantly differ (*P* > 0.05) in the absolute and relative testicular weights from the control group (Table 1).

### Testicular concentrations of Zn And/Or As after 60 consecutive days of treatment

The ZnONPs exposure in the ZnONPs group or ZnONPs + As group showed significant elevations in Zn level in the testicular tissues compared to the control group (Table 2). After co-administration of GA for ZnONPs and As-exposed male rats after 60 consecutive days from the beginning of the experiment, there was a significant decrease (*P* < 0.05) in testicular Zn amount than groups exposed to ZnONPs combined with As (Table 2).

**Table 2:** Effect of gallic acid (GA) on serum levels of male hormones and testicular oxidative stress indicators and testicular tissue content of arsenic (As) and zinc of adult male Sprague Dawley rats exposed to zinc oxide nanoparticles (ZnONPs) and/or arsenic trioxide (As) for 60 days

Estimated parameters		Experimental groups				
	Control	GA	ZnONPs	As	ZnONPs + As	ZnONPs + As + GA
Testosterone (ng/mL)	0.93 ^a^ ±0.14	0.93 ^a^ ±0.15	0.53 ^b^ ±0.10	0.18 ^c^ ±0.04	0.13 ^c^ ±0.02	0.73 ^ab^ ±0.08
Estradiol (pg/mL)	12.80 ^c^±0.78	15.03 ^c^±0.95	28.07 ^b^ ±6.04	34.80 ^b^ ±1.11	44.50 ^a^ ±0.54	18.40 ^c^ ±1.08
LH (ng/mL)	2.43 ^a^ ±0.10	2.70 ^a^ ±0.32	1.76 ^bc^ ±0.26	1.77 ^bc^ ±0.16	1.33 ^c^ ±0.17	2.10 ^ab^ ±0.04
FSH (ng/mL)	3.23 ^b^ ±0.06	3.97 ^a^ ±0.08	2.71 ^cd^ ±0.19	2.97 ^bc^ ±0.22	2.50 ^d^ ±0.15	3.13 ^bc^ ±0.10
SOD (U/mL)	44.66 ^a^±1.48	48.83 ^a^±2.70	17.19 ^c^ ±1.61	8.29 ^d^ ±1.00	16.17 ^c^ ±0.77	36.61 ^b^ ±1.70
GPx (pg/mL)	119.35^ab^±2.12	137.49^a^±2.95	85.27^cd^±12.02	64.65^d^±12.23	41.02 ^e^±1.49	101.56 ^bc^±2.10
MDA (nmol/mL)	130.01^b^ ±3.28	93.91 ^c^ ±4.71	133.75^b^ ±4.29	150.77^a^±3.92	157.78^a^±4.96	124.74 ^b^ ±2.65
As residues (ppm)	ND	ND	ND	0.25 ^a^ ±0.01	0.16 ^b^ ±0.02	0.08 ^c^ ±0.00
Zn residues (ppm)	19.60 ^c^ ±0.59	13.80 ^e^ ±0.31	23.80 ^b^ ±0.39	14.60 ^e^ ±0.78	39.18 ^a^ ±0.51	16.88 ^d^ ±0.64

*LH*, luteinizing hormone; *FSH*, follicle-stimulating hormone; *SOD*, super oxide dismutase; *GPx*, glutathione peroxidase; *MDA*, malondialdehyde; *Zn*, Zinc. Means within same row carrying different superscripts are significant different at* p* < 0.05. Values shown are means ± SE. *n* = 10 group

Concerning As residues, rats subjected to As merely or with ZnONPs showed a significant increase in testicular As concentration after 60 consecutive days from the experiment’s beginning compared with the control group, as presented in (Table 2). The co-administration of GA for As and ZnONPs intoxicated male rats after 60 consecutive days from the beginning of the experiment revealed a marked decrease (*P* < 0.05) in testicular As residue concentration compared with rats exposed to As and ZnONPs as presented in Table 2.

### Effects on semen analysis

The individual GA administration resulted in a significant (*P* < 0.05) rise in sperm count (9.22%) with a significant (*P* < 0.05) decrease in atypical sperm percentage (25.47%) when compared to the control group (Table 1). Compared to the control groups, the ZnONPs, As, and ZnONPs+As groups emerged with a significant (*P* < 0.05) drop in the proportion of motile spermatozoa by 47.76%, 44.03%, and 53.35%, and in the sperm cell counts by 19.43%, 18.45%, and 50.97%, respectively, than the control groups as shown in Table 1. The more pronounced reduction was identified in the ZnONPs+As co-exposed group compared to individual treatment with ZnONPs or As.

**Fig. 2:**
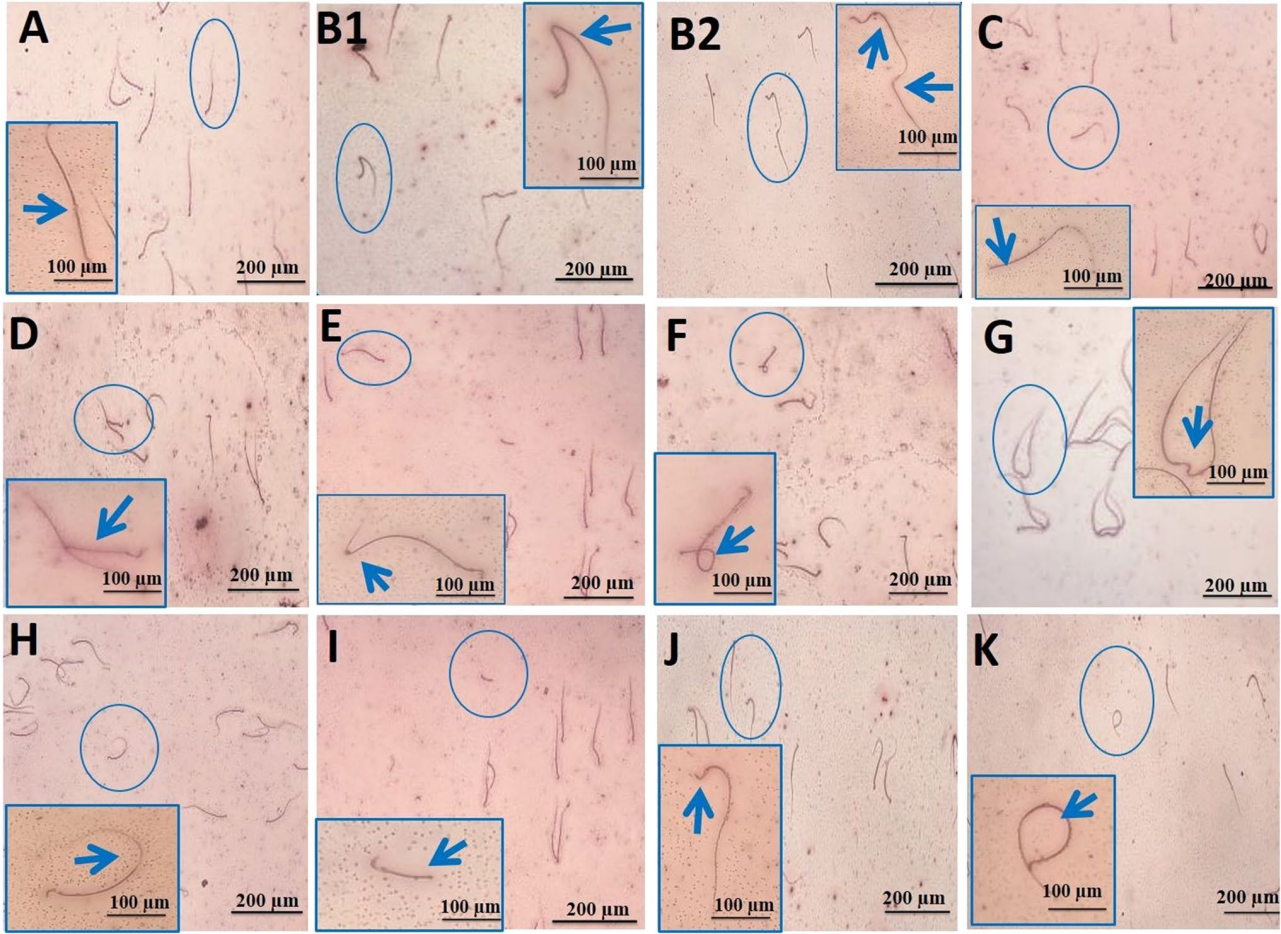
Morphological changes in rat sperm following oral exposure to zinc oxide nanoparticles and/or arsenic. (**A**) Normal sperm, (**B**1 and **B**2) Bent tail, (**C**) Detached head, (**D**) Double head with fused tail, (**E**) Bent tail with protoplasmic droplet, (**F**) Coiled tail, (**G**) Fused heads, (**H**) Curved tail, (**I**) Short tail, (**J**) Bent head, (**K**) Looped tail

The significant (*P* < 0.05) increment in abnormal sperm percentage was detected at 100 mg/kg bwt of ZnONPs (91.88%) and at 8 mg/kg of As (88.29%) administered merely and in combination (133.88%) than the control group. The ZnONPs+As co-exposed group displayed significant (*P* < 0.05) elevation in abnormal sperm percentage than the ZnONPs or As group (Table 1 and Fig. 2). In comparison to the control group, the administration of GA in combination with ZnONPs and As considerably (*P* < 0.05) reduced decreases in spermatozoa motility (25.37%), sperm cell concentrations (9.71%), and aberrant spermatozoa percentage (61.76%). GA was remarkably protective against spermiogram disruptions caused by ZnONPs and/or As.

### Effects on serum reproductive hormones

In comparison to the control group, the ZnONPs (43.01%), As (80.65%), and ZnONPs+As (86.02%) groups showed a substantial drop (*P*<0.05) in serum testosterone levels (Table 2). The more pronounced (*P* < 0.05) decrease in serum testosterone level was in the ZnONPs+As co-exposed group. The serum testosterone concentration of the ZnONPs+As+GA-treated group was significantly (*P* < 0.05) higher than that of the ZnONPs+As group and virtually equal to that of the control group.

The concentration of serum FSH was found to be considerably (*P* < 0.05) lower in the groups treated with ZnONPs (16.1%), As (8.05%), and ZnONPs + As (22.60%), compared to the control group. The reduction in serum FSH hormone level was more evident in the co-exposed group with ZnONPs and As. GA administered alone or co-administered with As and ZnONPs ameliorated (*P* < 0.05) the serum FSH level to be 22.91% and 3.1%, respectively, relative to control values (Table 2).

Serum LH level decreased significantly (*P* < 0.05) in the ZnONPs (27.57%), As (27.16%), and ZnONPs+As (45.27%) exposed rats than the control rats. A significant decline (*P* < 0.05) in serum LH hormone level was observed in the group exposed to ZnONPs+As. The serum LH content in the ZnONPs+As+GA-treated group was 13.58% higher than in the ZnONPs+As group, and this difference was statistically significant (*P* < 0.05) when normalized to the control value (Table 2).

As shown in Table 2, serum estradiol levels increased significantly (*P* < 0.05) in the ZnONPs (119.30%), As (171.88%), and ZnONPs+As (247.66%) groups than the control group. The more obvious increase (*P* < 0.05) in serum estradiol level was in the ZnONPs+As co-exposed group. There was a (*P* < 0.05) significant reduction (43.75%) in serum estradiol in the ZnONPs+As+GA group than the ZnONPs+As group and very close to the control group.

### Changes in testicular oxidative status

Relative to the control groups, the ZnONPs, As, and ZnONPs+As groups emerged with a significant (*P* < 0.001) drop in SOD concentration by 61.51%, 81.44%, and 63.79%, respectively. Both ZnONPs and ZnONPs + As groups revealed significant (*P* < 0.05) elevations in SOD concentration compared with the As group. Testicular GPx levels significantly (*P* < 0.05) reduced in the ZnONPs (28.55%), As (45.83%), and ZnONPs+As (65.63%) groups than the control group (Table 2). Both testicular GPx and SOD concentrations significantly (*P* < 0.05) restored to 14.91% and 18.03% in the ZnONPs+As+GA-treated group than the control group. The testicular MDA level was significantly (*P* < 0.05) reduced (27.77%) in the GA group compared to the control group. MDA in testes increased significantly (*P*<0.05) in the ZnO-NPs (2.88%), As (15.97%), and ZnONPs+As (21.36%) groups than the control group (Table 2). The more evidenced increase in MDA level was noticed in the co-administered group As with ZnONPs compared with individual administration for each. The MDA testicular content significantly (*P* < 0.05) reduced (4.05%) in the ZnONPs+As+GA group compared to the combined group, and reestablished closely to the control value.

### Histopathological findings

#### Testis

**Fig. 3:**
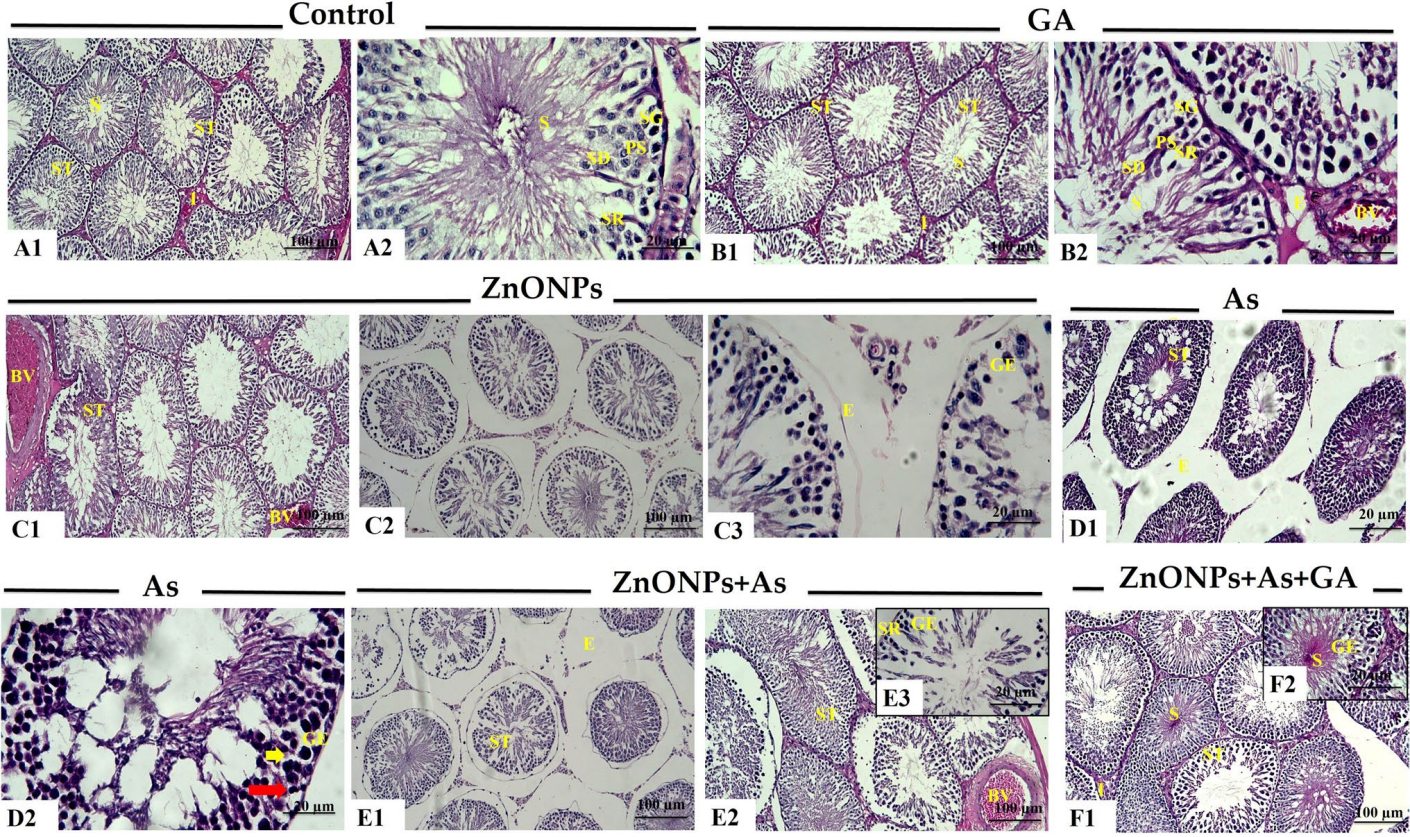
Representative histopathological photomicrographs of hematoxylin-stained cross-sections of rat testes. Control group (A1 and A2), gallic acid (GA)-treated group (B1 and B2), zinc oxide nanoparticles-exposed group (ZnONPs) (C1, C2, and C3), arsenic trioxide (As) -exposed group (D1 and D2), zinc oxide nanoparticles and arsenic trioxide (ZnONPs+As) co-exposed group (E1, E2, and E3), and zinc oxide nanoparticles and arsenic trioxide co-exposed group treated with gallic acid (ZnONPs+As+GA) (F1, and F2). Disorganized germinal epithelium **(thick short arrow),** dark pyknotic nuclei **(red arrow),** interstitial edema (E), seminiferous tubules (ST), spermatozoa (S), spermatogonia (SG), primary spermatocyte (SD), Sertoli cells (SR), and vacuolation **(yellow arrow)**

In the control group, there were no histological alterations. The sections showed typical seminiferous tubules with germinal epithelium lining the lumen and spermatozoa. The germinal epithelium had spermatogenic cells and Sertoli cells. Mature spermatozoa, spermatids, spermatocytes, and spermatogonia are regularly organized. Sertoli cells were identified between spermatogenic cells as pyramidal cells (Fig. 3A). Also, the testis of GA-treated rats displayed normal structure without any pathological alterations except for some blood vessel congestion and edema in some sections (Fig. 3B). Conversely, tissue sections from ZnONPs or As groups showed many histopathological alterations. The latter was observed as atrophied seminiferous tubules with an asymmetrical outline and a detached and thickened basement membrane. Other tubules revealed disturbed spermatogenesis with the loss of layers of germinal epithelium. Some tubules exhibited epithelium with darkly stained pyknotic nuclei and vacuolations, which were more prominent in the As-treated group. Sertoli cells appeared necrosed. There was deposition of acidophilic material and edema in interstitial tissue. Also, severe congestion of the sub-capsular and interstitial blood vessels with thickening in the wall with vacuolations was seen in the ZnONPs-treated group. There was a thickened capsule (Fig. 3C and D). The histopathological alterations increased in severity in the group co-exposed to ZnONPs and As (Fig. 3E). Conversely, the microscopic examination of co-exposure ZnONPs and As with GA-treated rats revealed an improved histological picture. It showed a normal structure with active spermatogenesis. Few tubules showed epithelial vacuolations (Fig. 3F).

#### Prostate glands

**Fig. 4:**
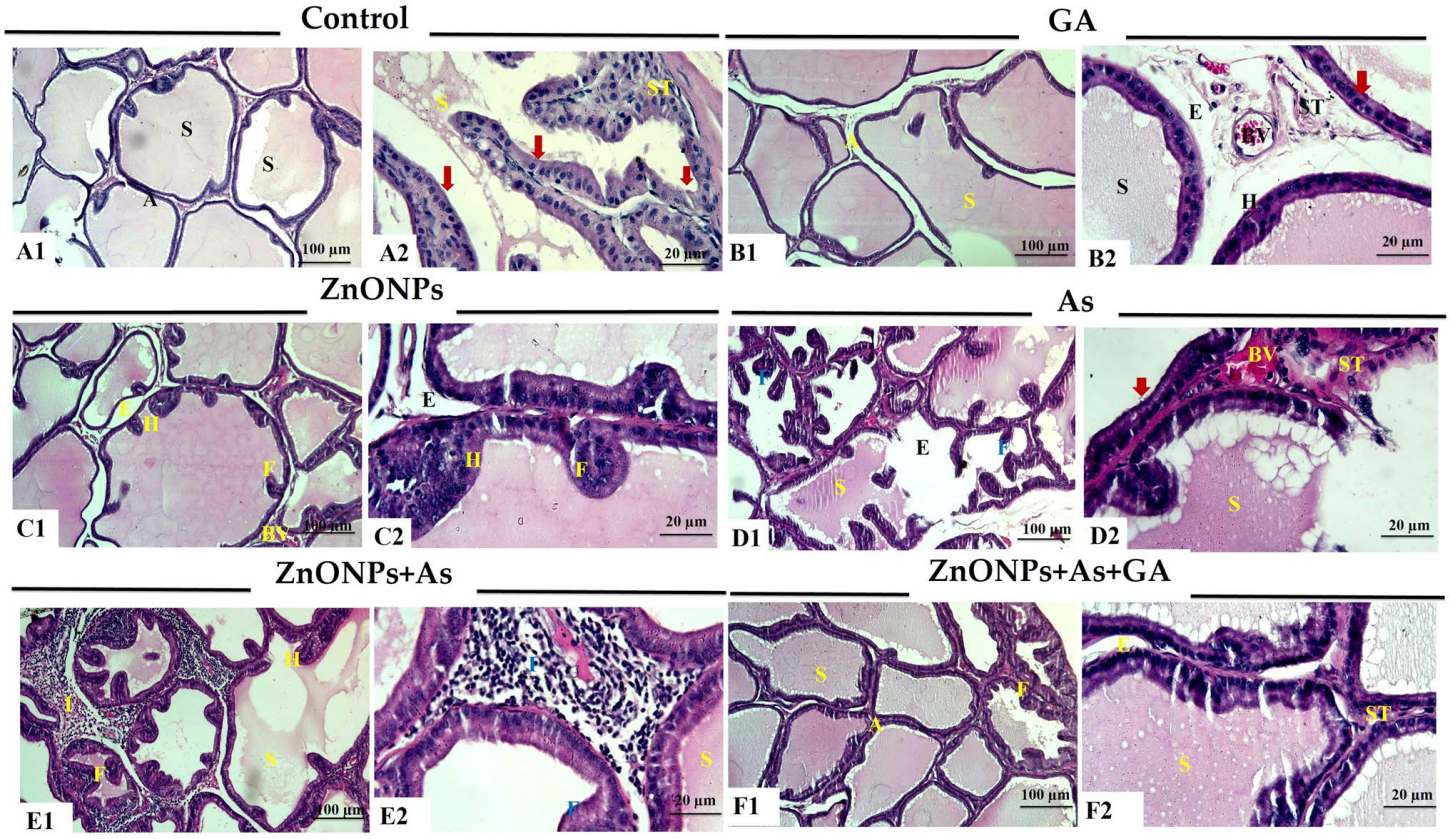
Representative histopathological photomicrographs of hematoxylin-stained cross-sections of rat prostate gland. Control group (A1 and A2), gallic acid (GA)-treated group (B1 and B2), zinc oxide nanoparticles (ZnONPs)-exposed group (C1 and C2), arsenic trioxide (As)-exposed group (D1 and D2), zinc oxide nanoparticles and arsenic trioxide (ZnONPs+As) co-exposed group (E1 and E2), and zinc oxide nanoparticles and arsenic trioxide co-exposed group treated with gallic acid (ZnONPs+As+GA) (F1 and F2). Acini (A), acinar hyperplasia (H), blood vessels (BV), edema (E), epithelial hyperplasia (H), fibromuscular stroma (ST), interstitial inflammatory cells (I) interstitial edema (E), papillary folds (F), prostatic secretions (S), simple columnar epithelium **(thick arrow)**, and fibromuscular stroma (ST)

The histological evaluation of the prostate glands of the control and GA groups exhibited normal structure. The gland consisted of tubuloalveolar acini aggregations of different sizes and shapes. The acini were lined with simple columnar or cuboidal cell epithelium. Prostatic secretions were seen inside their lumen. The acini implanted in the fibromuscular stroma enclosed blood vessels. An outer capsule surrounded them. In addition, some acini in the GA group had mild epithelial hyperplasia and blood vessel congestion (Fig. 4A and B). The prostate gland of ZnONPs or As-treated rats showed many pathological changes compared with the control group. Decreasing acinar size was seen, and some of the acini had papillary projections towards the lumen. The acini were lined with vacuolated flattened epithelium. There was damage in some acini. Additionally, their lumens included little vacuolated secretions. There was congestion of interstitial blood vessels. Interstitial edema was exhibited between the acini. There were inflammatory cells surrounding the acini that were monocellular. A markedly thick fibro-muscular layer was observed. These changes were more obvious in the group treated with As (Fig. 4C and D). Moreover, the group co-exposed to ZnONPs and As showed more severe pathological changes (Fig. 4E). On the contrary, ZnONPs+ As+ GA-treated group showed normal acini. Besides, the acinar lumen was approached by papillary projections in a few acini that had mild epithelial hyperplasia. Additionally, minor edema and congestion were noted (Fig. 4F).

#### Seminal vesicles

**Fig. 5:**
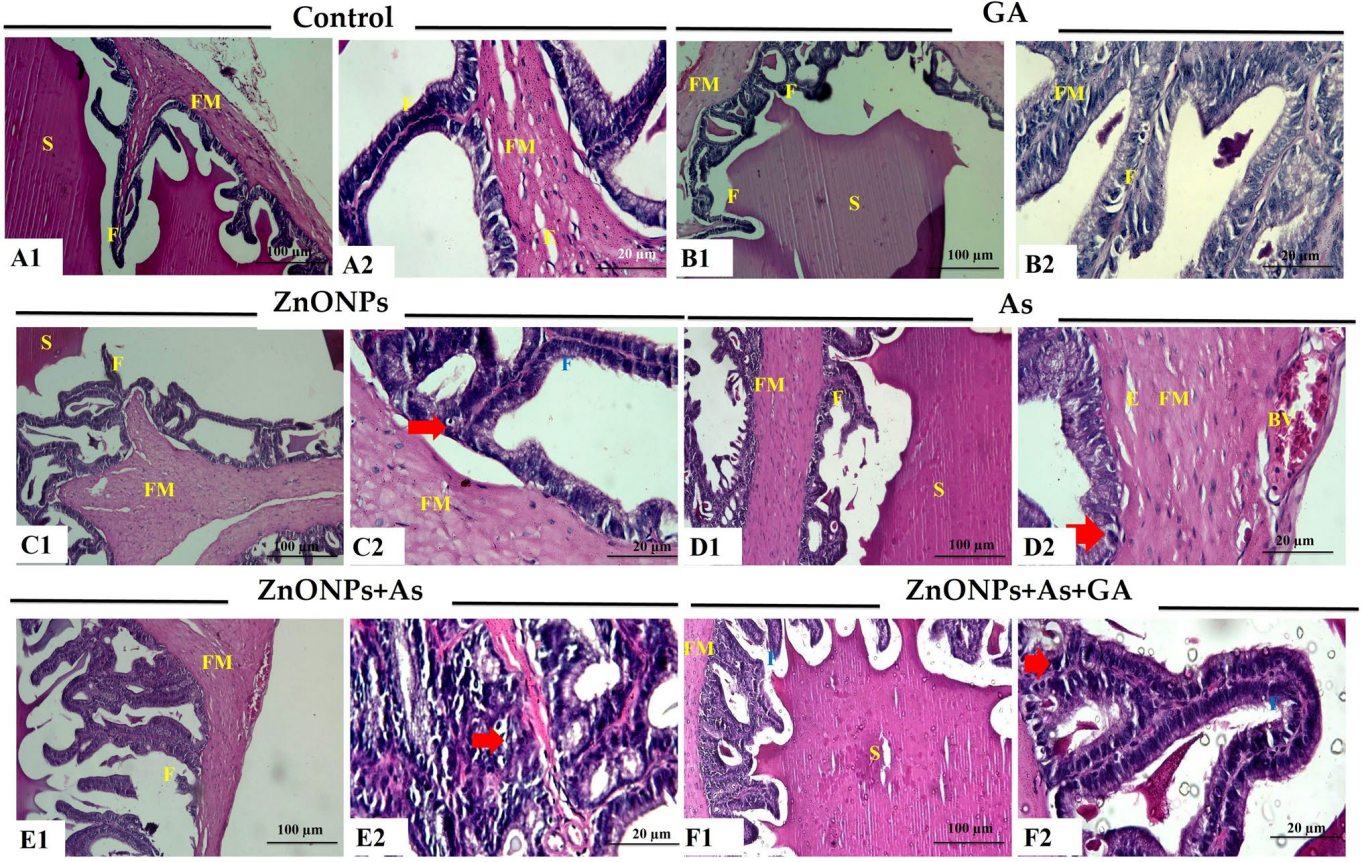
Representative histopathological photomicrographs of hematoxylin-stained cross-sections of rat Seminal vesicle. Control group (A1 and A2), gallic acid (GA)-treated group (B1 and B2), zinc oxide nanoparticles (ZnONPs)-exposed group (C1 and C2), arsenic trioxide (As)-exposed group (D1 and D2), zinc oxide nanoparticles and arsenic trioxide (ZnONPs+As) co-exposed group **(**E1 and E2), and zinc oxide nanoparticles and arsenic trioxide co-exposed group treated with gallic acid (ZnONPs+As+GA) **(**F1 and F2). Edema (E), fibro-muscular layer (FM), secretions (S), and vacuolated epithelium **(Arrows)**

Examining the seminal vesicles of the adult male rats’ control and GA groups showed a normal histological structure. Smooth muscle-enclosed branching convoluted mucosal folds. The pseudostratified columnar epithelium with foamy cytoplasm and extremely vesicular nuclei lines the folds. Pink secretion fills the lumen of the gland. The gland is encircled with fibro-muscular capsules and trabeculae pass through them. Mild vacuolations in some folds of epithelium in the GA group (Fig. 5A and B). While ZnONPs and/or As treatments affected the histological structure of the seminal vesicles of rats compared with the control group. Hyperplasic folds that were heavily coiled formed on their mucosal surfaces, which were significantly enlarged. Prominent vacuolated epithelium was seen. Some folds exhibited destruction. There was a lot of thickening, swelling, clogged blood vessels, vacuolations, and infiltrations of monocellular inflammatory cells in the fibro-muscular layer. Scanty secretions in the lumen of the acini were detected. Subcapsular edema and congestion were identified. The co-exposure to ZnONPs and As showed pathological alterations (Fig. 5C, D and E). On the other hand, ZnONPs and As with GA-treated rats showed normal structure seminal vesicles. Mild epithelial hyperplasia, papillary projections, edema, and congestion were observed (Fig. 5F).

### Histomorphometric results

As indicated in Table 3, the germinal epithelium height was significantly reduced in groups exposed to ZnONPs and/or As compared with the control group. In addition, seminiferous tubule diameters in the control and GA groups were nearly similar but significantly reduced in the ZnONPs and/or As groups. Tubular lumen diameter values in all experimental groups were significantly increased except for the GA group. Also, there was a significant increase in the tunica propria thickness in the treated groups, while the GA group significantly decreased. Testicular capsule thickness was greatly increased in the As group compared to the ZnONPs group and slightly augmented in the combination group.

**Table 3:** Effect of gallic acid (GA) on testicular morphometric analysis and immunoexpression of iNOS and PCNA of adult male Sprague Dawley rats exposed to zinc oxide nanoparticles (ZnONPs) and/or arsenic trioxide (As) for 60 days

	Control	GA	ZnONPs	As	ZnONPs + As	ZnONPs + As + GA
Epithelial height	49.67 ^a^ ±4.73	52.67 ^a^ ±2.72	36.00 ^b^ ±1.41	39.00 ^b^ ±0.82	16.00 ^c^ ±1.47	47.67 ^a^ ±2.59
Tubular diameter	282.00 ^a^ ±3.08	287.33 ^a^ ±4.55	240.00 ^bc^ ±1.22	229.33 ^c^ ±3.27	194.67 ^d^ ±13.12	257.00 ^b^ ±3.49
Lumen diameter	136.33 ^cd^ ±4.55	124.00 ^d^ ±3.74	180.33 ^b^ ±1.65	187.67 ^ab^ ±4.55	198.33 ^a^ ±6.13	141.33^c^ ±9.29
Tunica propria	2.33 ^bc^ ±0.24	1.67 ^c^ ±0.24	3.00 ^ab^ ±0.41	3.00 ^ab^ ±0.41	3.67 ^a^ ±0.24	2.67 ^abc^ ±0.47
Capsule	7.33 ^cd^ ±0.62	5.67 ^d^ ±0.24	17.67 ^b^ ±0.94	22.33 ^a^ ±2.66	24.33 ^a^ ±1.25	11.00 ^c^ ±1.22
iNOS immunoexpression area	8.80 ^c^ ±0.60	10.40 ^c^ ±0.76	21.20 ^b^ ±0.70	21.80 ^b^ ±0.87	41.00 ^a^ ±1.06	10.00 ^c^ ±0.58
PCNA immunoexpression area	12.20 ^a^ ±0.70	13.20 ^a^ ±0.87	5.40 ^c^ ±0.88	5.20 ^c^ ±0.48	4.60 ^c^ ±0.66	8.52 ^b^ ±0.32

*iNOS*, inducible nitric oxide synthase; *PCNA*, Proliferating cell nuclear antigen. Means within the same row carrying different superscripts are significantly different at* p* < 0.05. The values shown are means ± SE. *n* = 10

### Immunohistochemistry analysis

#### iNOS immunohistochemistry

**Fig. 6:**
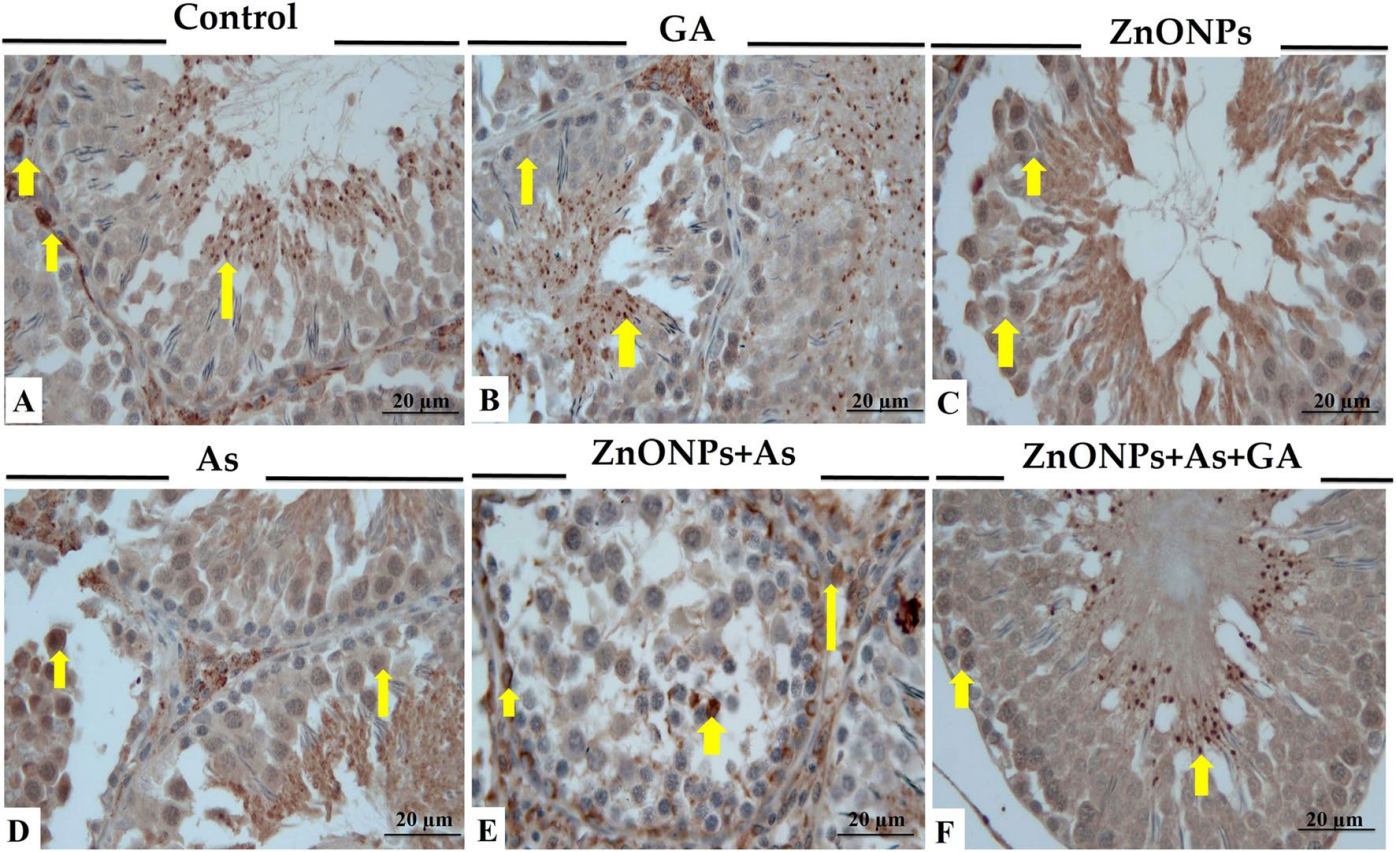
Representative histopathological photomicrographs of testicular tissue sections stained for iNOS immunoexpression. (**A**) Control group. (**B**) Gallic acid (GA)-treated group. (**C**) Zinc oxide nanoparticles (ZnONPs)-exposed group. (**D**) Arsenic trioxide (As)-exposed group. (**E**) Zinc oxide nanoparticles and arsenic trioxide (ZnONPs+As) co-exposed group. (**F**) Zinc oxide nanoparticles and arsenic trioxide co-exposed group treated with gallic acid (ZnONPs+As+GA). iNOS positive reactions in the cytoplasm of spermatogenic cells and Leydig cells are represented by a brown color (arrows)

There was an obvious variation in the staining intensity between testicular cells of different groups, as shown in Table 3. Control and GA rat testes showed a mild intensity of iNOS that was presented in the seminiferous tubules and Leydig cells. Expression of iNOS was represented by a brown color in the cytoplasm of cells (spermatogonia, spermatocytes, spermatids, Sertoli cells, and Leydig cells). Treatment with ZnONPs and/or As increased iNOS immunostaining intensity and distribution. They were variable intensity from moderate to intense. The increase in expression was more obvious in rats treated with ZnONPs and As than in the control rats. Yet, treatment with ZnONPs, As, and GA displayed mild iNOS staining intensity (Fig. 6). Moreover, the GA+ZnONPs+As-treated group displayed nearly similar iNOS immunoreactivity reactions to the control group.

#### PCNA immunohistochemistry

**Fig. 7:**
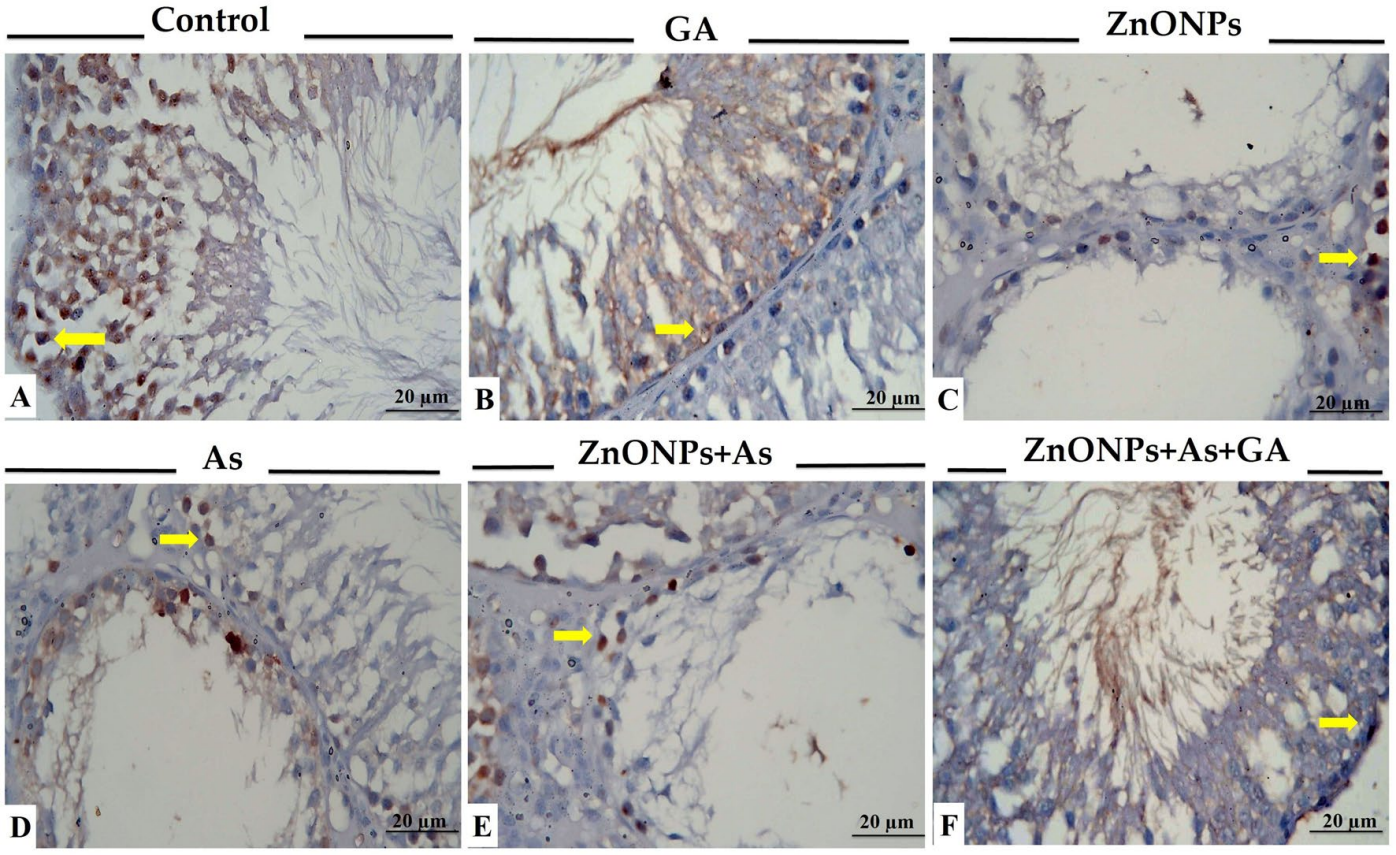
Representative histopathological photomicrographs of testicular tissue sectionsm stained for PCNA immunoexpression. (**A**) Control group. (**B**) Gallic acid (GA)-treated group. (**C**) Zinc oxide nanoparticles (ZnONPs)-exposed group. (**D**) Arsenic trioxide (As)-exposed group. (**E**) Zinc oxide nanoparticles and arsenic trioxide (ZnONPs+As) co-exposed group. (**F**) Zinc oxide nanoparticles and arsenic trioxide co-exposed group treated with gallic acid (ZnONPs+As+GA). PCNA-positive reactions in the cytoplasm of spermatogenic cells and Leydig cells are represented by a brown color (arrows)

In the control and GA groups, spermatogonia exclusively reacted positively to PCNA, but the other spermatogenic cell types did not. Also, Sertoli, Leydig, and sperm cells negatively reacted to PCNA. In treated groups, a weak positive reaction was observed in spermatogenic cells as brown. Some of the Sertoli cells in the seminiferous tubules and Leydig interstitial cells of ZnONPs, together with As-treated group rats, displayed a weak reaction for PCNA than the control group (Fig. 7). In addition, there was a significant decrease in PCNA expression in the testes of ZnONPs and/or As group-treated rats than the control group. Yet, groups treated with GA were nearly comparable to the control group (Table 3).

## Discussion

The final weight and weight gain of the rats in the rat subjected to a 60-day oral administration of the ZnONPs and/or As were significantly decreased than the control group, with a marked reduction in the ZnONPs+As group. The decrease in body weight with ZnONPs treatment could be attributed to feed consumption loss (Srivastav et al. 2016). Arsenic-associated body weight reduction indicated that the animals’ metabolic state was severely affected (Guvvala et al. 2019). Yet, a significant re-establishment of the body weight was recorded in ZnONPs+As-exposed rats with GA treatment. This could be related to the reported beneficial role of GA on intestinal health and metabolic enzymes (Maheshwari et al. 2023).

In various toxicity studies, reproductive organ index weight was found to be a key reproductive health indicator (Hassan et al. 2021; Behairy et al. 2022; Mostafa-Hedeab et al. 2023). This study found that rats given ZnONPs had significantly higher gonado-somatic indices than the control group. This finding could be due to edema and congestion, as confirmed by histological examination of testicular tissues. In contrast, rats given As alone or in combination with ZnONPs had a significant drop in testicular weight, which could be ascribed to the diminished germ cell maturity and the decline in the width of the seminiferous tubules due to rising oxidative stress (Olfati and Tvrda 2021). Yet, the recovery of weight gain and testicular weight in ZnONPs+As+GA-treated rats demonstrated that GA improves metabolic processes and reproductive organ health (Owumi et al. 2020).

This study found that ZnONPs and ZnONPs+As had greater testis Zn content, which may indicate that ZnONPs have crossed the BTB. Metal NPs can get through the BTB due to their size, triggering inflammation that compromises the BTB’s integrity (Vassal et al. 2021). In this regard, ZnONPs exposure has been reported to elevate ROS generation and reduce tight junction proteins which aid in the adhesion of Sertoli cells forming the BTB, and so accumulate in the testis (Han et al. 2016). Yet, the co-treatment with GA minimized Zn and As residues in testicular tissues due to the GA antioxidant effects. Moreover, Rocha et al. (2019) verified that GA cytoprotection is associated with the chelating activity verified by infrared spectroscopy.

Herein, both ZnONPs and As caused a detrimental impact on sperm quantity and quality, reducing the sperm cell numbers and motility while elevating the number of morphological abnormalities. Besides, Deore et al. (2021) reported that ZnONPs exposure reduced mitochondrial membrane potential, which is a key component of energy storage during oxidative phosphorylation and aids in sperm movement and viability. Moreover, As exposure causes dysregulation of multiple proteins responsible for spermatogenesis (Barbhuiya et al. 2021). Furthermore, mammalian sperm contain an abundance of thiol-rich protamines in their nuclear chromatin, as well as a sulfhydryl group in the sperm flagellum, both of which are believed to be critical to sperm stability and motility support (Renu et al. 2018; Behairy et al. 2020). As is known to be a thiol inhibitor and ROS generator; a decline in motility and its particular characteristics could be due to an elevated amount of As in the epididymis, where sperm is undergoing maturation and gains motility (Abdulkadhar et al. 2014). On the contrary, GA treatment significantly increased sperm count, motility, and normal morphology of sperm cells compared to the ZnONPs+As group. GA could boost the count and spermatogenesis index in the treated rats by enhancing the body’s anti-oxidant protection (Abarikwu et al. 2014). GA has the potential to boost ATP production, which in turn enhances sperm motility and viability (Hosseinzadeh and Mehrzadi 2022). Besides, GA could enhance sperm motility by raising intracellular calcium levels and allowing cyclic adenosine monophosphate analogs to get through membranes and inhibit phosphodiesterase (Güngör et al. 2019).

In this study, ZnONPs and/or As exposure significantly increased serum estradiol relative to the control group while considerably decreasing serum testosterone, FSH, and LH. The reductions in serum levels of testosterone by ZnONPs were explained by the reduced expression of steroidogenic proteins in testes and the raised apoptosis in Leydig cells in the study of Pinho and Rebelo (2020). Besides, As metabolites have been reported to affect the hypothalamic-pituitary axis and LH and FSH plasma concentrations, affecting Leydig cell function and testosterone production (Kim and Kim 2015). Additionally, the elevation of estradiol hormone in the ZnONPs and/or As exposed rats could be a negative feedback mechanism to the increased testosterone (Stephens-Shields et al. 2022). However, the recovery of serum levels of testosterone, LH, FSH, and estradiol in rats received ZnONPs, As, and GA demonstrated the protective impact of GA against ZnONPs and/or As-mediated hormonal deficiency in the experimental rats. GA might promote the hypothalamic-pituitary gonadal axis, which augments testicular function and increases testosterone production (Jalili et al. 2020).

Herein, individual ZnONPs and co-exposure with As altered rats’ testes’ redox pathway, causing significant SOD and GPx alterations and increased MDA. ROS excessive production and cellular oxidative stress are the most prevalent outcomes of NP-cell interaction and the primary mechanism of toxicity (Sharma et al. 2012). On the other side, As causes the formation of ROS, increases free radicals, and reduces the activity of thiol group-rich antioxidants like GSH (Rachamalla et al. 2022). On the contrary, the safeguarding impact of GA on oxidative stress-induced testicular harm results from either direct antioxidant activity or stimulation of antioxidant enzyme activity (Bello and Idris 2018). Furthermore, the positive effect of GA can be attributed to its ability to induce the secretion of the metallothionein enzyme which is responsible for stimulating the GSH synthetic pathway (Ali et al. 2021).

In the current study, ZnONPs and/or As exposure increased the intensity of iNOS immunostaining compared to the control rats. Because of the constant irritation of cells caused by oxidative stress, ZnONPs exposure may result in inflammation in the reproductive organs (Mesallam et al. 2019). As also triggers iNOS, resulting in greater NO production, which collaborates with SOD to create the powerful cytotoxic agent peroxynitrite (Wang et al. 2013). The main impact of peroxynitrite is the nitration of cellular proteins, which leads to nitrosative stress and exhaustion of endogenous antioxidant defense systems (Bartesaghi and Radi 2018). Yet, the current findings of this study showed that GA has an anti-inflammatory property that reduces ZnONPs and/or As-induced stimulation of iNOS expression. GA inhibits inflammatory processes by hindering the NF-B pathway, which results in the down-regulation of pro-inflammatory cytokines (Hosseinzadeh et al. 2019). Additionally, in this study, PCNA, a key replication factor, was incorporated as a biomarker to assess spermatogenesis (Elashal et al. 2024). Herein, PCNA-positive testicular germ cells were considerably reduced in rats exposed to ZnONPs and/or As, indicating a disruption in proliferation and spermatogenesis (Nassar et al. 2017). Yet, the increased expression of PCNA in ZnONPs + As+ GA-treated testis in the current study demonstrates the ability of this phenolic substance to neutralize the antiproliferative activity of ZnONPs+As.

## Conclusion

**Fig. 8:**
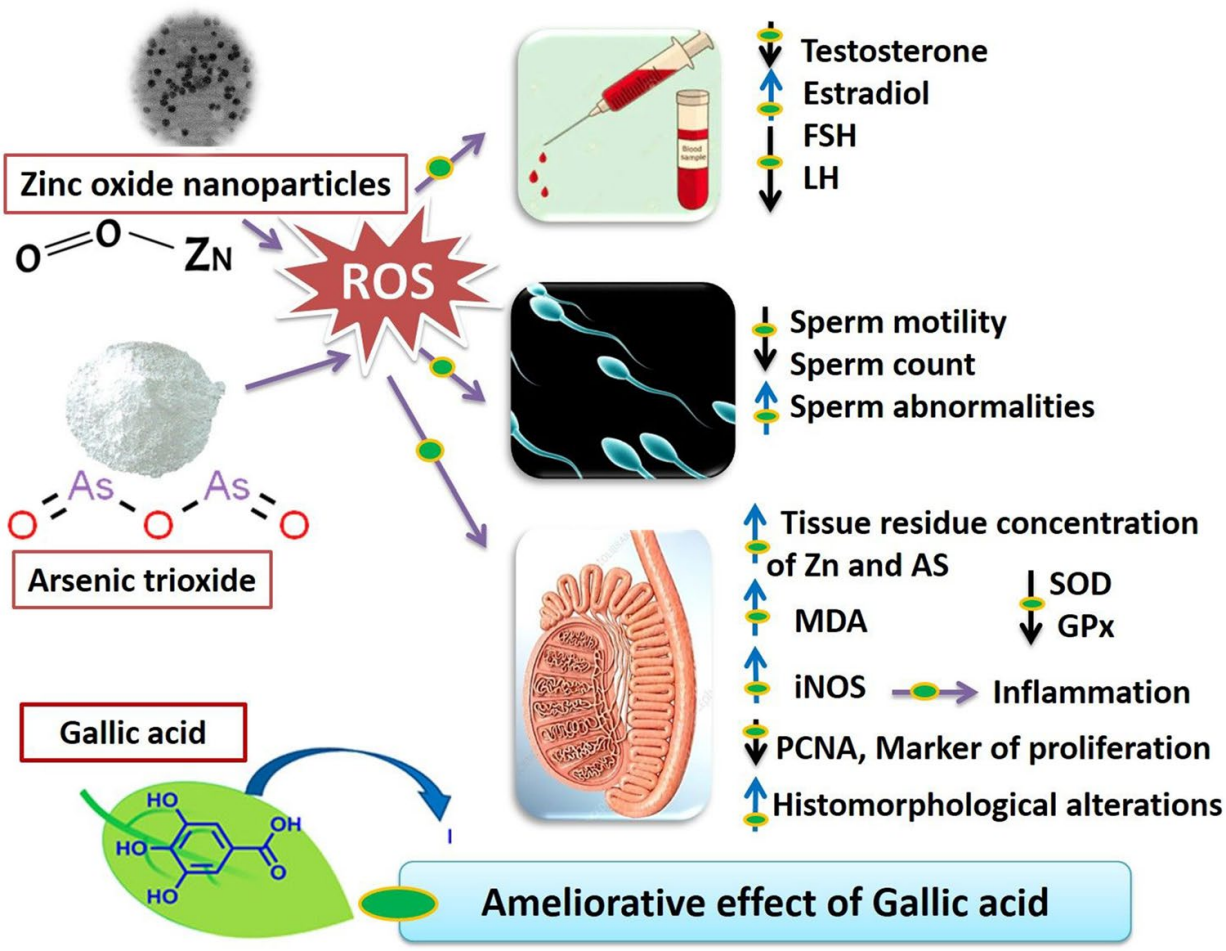
Proposed mechanism of protective effect of gallic acid (GA) against zinc oxide nanoparticles (ZnONPs) and arsenic (As) reproductive toxicity

This study concluded that exposure to ZnONPs and As, either individually or in combination, resulted in testicular toxicity characterized by alterations in sperm characteristics, reproductive hormones, oxidant/antioxidant status, and testicular morphometric analysis. Moreover, the combined exposure exhibited the highest frequency of reproductive function alterations (Fig. 8). Moreover, the present study demonstrated the potential advantages of GA in mitigating the adverse effects on reproductive parameters induced by ZnONPs and As in rats probably via its anti-oxidative and anti-inflammatory activities. Consequently, GA oral dosing as a dietary supplement or intervention strategy could be proposed as a potential approach to counteract the adverse effects of environmental toxicants on reproductive health.

## Data Availability

Data is provided within the manuscript.
